# 2-*O*-α-D-Glucosylglycerol Phosphorylase from *Bacillus selenitireducens* MLS10 Possessing Hydrolytic Activity on β-D-Glucose 1-Phosphate

**DOI:** 10.1371/journal.pone.0086548

**Published:** 2014-01-22

**Authors:** Takanori Nihira, Yuka Saito, Ken’ichi Ohtsubo, Hiroyuki Nakai, Motomitsu Kitaoka

**Affiliations:** 1 Graduate School of Science and Technology, Niigata University, Niigata, Japan; 2 National Food Research Institute, National Agriculture and Food Research Organization, Tsukuba, Ibaraki, Japan; University of Potsdam, Germany

## Abstract

The glycoside hydrolase family (GH) 65 is a family of inverting phosphorylases that act on α-glucosides. A GH65 protein (Bsel_2816) from *Bacillus selenitireducens* MLS10 exhibited inorganic phosphate (Pi)-dependent hydrolysis of kojibiose at the rate of 0.43 s^−1^. No carbohydrate acted as acceptor for the reverse phosphorolysis using β-d-glucose 1-phosphate (βGlc1*P*) as donor. During the search for a suitable acceptor, we found that Bsel_2816 possessed hydrolytic activity on βGlc1*P* with a *k*_cat_ of 2.8 s^−1^; moreover, such significant hydrolytic activity on sugar 1-phosphate had not been reported for any inverting phosphorylase. The H_2_^18^O incorporation experiment and the anomeric analysis during the hydrolysis of βGlc1*P* revealed that the hydrolysis was due to the glucosyl-transferring reaction to a water molecule and not a phosphatase-type reaction. Glycerol was found to be the best acceptor to generate 2-*O*-α-d-glucosylglycerol (GG) at the rate of 180 s^−1^. Bsel_2816 phosphorolyzed GG through sequential Bi-Bi mechanism with a *k*_cat_ of 95 s^−1^. We propose 2-*O*-α-d-glucopyranosylglycerol: phosphate β-d-glucosyltransferase as the systematic name and 2-*O*-α-d-glucosylglycerol phosphorylase as the short name for Bsel_2816. This is the first report describing a phosphorylase that utilizes polyols, and not carbohydrates, as suitable acceptor substrates.

## Introduction

Phosphorylases catalyze the cleavage of glycosidic bonds through substitution with phosphate [1], [2]. These enzymes reversibly phosphorolyze glycosides to form corresponding monosaccharide 1-phosphates with retention or inversion of anomeric configuration. The sole exception is maltosyl-transferring starch synthase (EC 2.4.99.16) that generates maltose 1-phosphate [3]. All the phosphorylases reported (Table 1) exhibit strict substrate specificity in the phosphorolysis and regioselectivity in the reverse phosphorolysis. Phosphorylases have been classified in one of the following families in the Carbohydrate-Active Enzymes database (http://www.cazy.org/) based on the amino acid sequence similarity [4]: glycoside hydrolase families (GH) 13, 65, 94, 112, and 130, and glycosyltransferase families (GT) 4 and 35. Among these families, GH65, 94, 112, and 130 primarily comprises of phosphorylases that mainly catalyze the reversible phosphorolysis of α-glucosides, β-glucosides, β-galactosides, and β-mannosides, respectively, with inversion of anomeric configuration.

**Table 1:** List of known phosphorylases.

EC	name	mechanism	product	Family
**2.4.1.1**	glycogen/starch phosphorylase	retention	αGlc1*P*	GT35
**2.4.1.7**	sucrose phosphorylase	retention	αGlc1*P*	GH13
**2.4.1.8**	maltose phosphorylase	inversion	βGlc1*P*	GH65
**2.4.1.20**	cellobiose phosphorylase	inversion	αGlc1*P*	GH94
**2.4.1.30**	1,3-β-oligoglucan phosphorylase	inversion	αGlc1*P*	NI*
**2.4.1.31**	laminaribiose phosphorylase	inversion	αGlc1*P*	GH94
**2.4.1.49**	cellodextrin phosphorylase	inversion	αGlc1*P*	GH94
**2.4.1.64**	trehalose phosphorylase (inverting)	inversion	βGlc1*P*	GH65
**2.4.1.97**	β-1,3-glucan phosphorylase	inversion	αGlc1*P*	NI*
**2.4.1.211**	1,3-β-galactosyl-*N-*acetylhexosamine phosphorylase	inversion	αGal1*P*	GH112
**2.4.1.216**	trehalose 6-phosphate phosphorylase	inversion	βGlc1*P*	GH65
**2.4.1.230**	kojibiose phosphorylase	inversion	βGlc1*P*	GH65
**2.4.1.231**	trehalose phosphorylase (retaining)	retention	αGlc1*P*	GT4
**2.4.1.247**	1,4-β-d-galactosyl-l-rhamnose phosphorylase	inversion	αGal1*P*	GH112
**2.4.1.279**	nigerose phosphorylase	inversion	βGlc1*P*	GH65
**2.4.1.280**	*N*,*N'*-diacetylchitobiose phosphorylase	inversion	αGlcNAc1*P*	GH94
**2.4.1.281**	1,4-β-mannosyl-glucose phosphorylase	inversion	αMan1*P*	GH130
**2.4.1.282**	1,3-α-d-glucosyl-l-rhamnose phosphorylase	inversion	βGlc1*P*	GH65
**2.4.1.x**	β-1,4-mannooligosaccharide phosphorylase	inversion	αMan1*P*	GH130
**2.4.1.x**	1,4-β-mannosyl-*N*-acetylglucosamine phosphorylase	inversion	αMan1*P*	GH130
**2.4.1.x**	cellobionic acid phosphorylase	inversion	αGlc1*P*	GH94
**2.4.99.16**	starch synthase (maltose-transferring)	retention	αMal1*P*	GH13

* Not identified.

Various oligosaccharides have been synthesized by phosphorylases using the reverse phosphorolysis from the corresponding sugar 1-phosphate as a donor substrate and suitable carbohydrate acceptors [1], [2], [5], [6]. In addition, the reversibility allows the practical synthesis of oligosaccharides from the readily available natural sugars by using a single phosphorylase [7], [8], by the combined reaction of two phosphorylases that share the same sugar 1-phosphate or utilizing the compatible ones [1], [9]–[13], or by the combined reaction of two phosphorylases producing different sugar 1-phosphates with additional enzymes to convert the sugar 1-phosphates [14]–[16].

However, the utilizations of phosphorylases for the practical syntheses of oligosaccharides are limited by the fact that there is little variation among the phosphorylases, with only 22 known activities (Table 1). Therefore, it is of interest to identify phosphorylases with other substrate specificities. The following new phosphorylases have been reported within the past five years: GH13 maltosyl-transferring starch synthase (EC 2.4.99.16) [3], GH65 nigerose phosphorylase (EC 2.4.1.279) [17], GH65 3-*O*-α-d-glucosyl-l-rhamnose phosphorylase (EC 2.4.1.282) [18], GH94 cellobionic acid phosphorylase [19], GH112 4-*O*-β-d-galactosyl-l-rhamnose phosphorylase (EC 2.4.1.247) [20], GH130 β-1,4-d-mannosyl-d-glucose phosphorylase (EC 2.4.1.281) [21], GH130 β-1,4-mannooligosaccharide phosphorylase (EC 2.4.1.x) [22], and GH130 4-*O*-β-d-mannosyl-*N*-acetyl-d-glucosamine phosphorylase (EC 2.4.1.x) [23].

*Bacillus selenitireducens* is an anaerobic haloalkaliphilic bacterium that is often isolated from the bottom sediments [24]. It grows optimally in a salinity of 1.5 M NaCl. The salt-dependent proteins isolated from this bacterium, such as the halophilic extracellular arsenate reductase that shows its optimal activity at 2.5 M NaCl, can be adapted to function in extreme environments [25]. The genomic sequence of the *B. selenitireducens* MLS10 strain, which was isolated from the anoxic mud of a saline lake, has been published (GenBank accession No. CP001791.1). *B. selenitireducens* MLS10 possesses three genes encoding possible inverting phosphorylases belonging to GH65. We have revealed the activities of two of them: one (locus: Bsel_2056) is a maltose phosphorylase that produces derivatives of maltose possessing a branch at position 2 [26], and the other (locus: Bsel_1207) is a potassium ion-dependent trehalose phosphorylase [27]. In this study, we identified the specificity of the third GH65 protein (locus Bsel_2816, GenBank accession No. ADI00307.1) and found that it is unique among the known phosphorylases.

## Materials and Methods

### Materials

β-d-Glucose 1-phosphate (βGlc1*P*) disodium salt was purchased from Tokyo Chemical Industry (Tokyo, Japan). β-Phosphoglucomutase (βPGM), βGlc1*P* bis(cyclohexylammonium) salt, and d-glucose 1,6-bisphosphate (Glc16b*P*) were obtained from Sigma-Aldrich Chemicals (St. Louis, MO). Kojibiose, glucose 6-phosphate dehydrogenase (G6PDH, from *Leuconostoc mesenteroides*), thio-NAD^+^, and Glucose C-II Test Wako were purchased from Wako Pure Chemicals (Osaka, Japan). All other chemicals used are of reagent grade.

### Amino Acid Sequence Analysis

Multiple alignments of the amino acid sequences of GH65 proteins with known activities with that of Bsel_2816 were performed using the ClustalW version 2.1 on the DDBJ sever (http://clustalw.ddbj.nig.ac.jp/index.php?lang=ja). A phylogenetic tree was drawn from the results obtained with the neighbor-joining method using a TreeView version 1.6.6 (http://taxonomy.zoology.gla.ac.uk/rod/rod.html).

### Cloning of *bsel_2816*

A gene (GenBank ID: ADI00307.1) encoding Bsel_2816 was amplified by PCR from the genomic DNA of *B. selenitireducens* used as template and KOD-plus DNA polymerase (Toyobo, Osaka, Japan) with the following primers based on the genomic sequence: 5′-aaaccatgggccatgaaattggagaacatc-3′ as the forward primer containing the *Nco*I site (underlined) and 5′-tttctcgaggcgggacttggtgatgcg-3′as the reverse primer containing the XhoI site (underlined). The amplified gene was purified using the FastGene Gel/PCR Extraction Kit (Nippon Genetics Co., Ltd., Tokyo, Japan), digested by NcoI and XhoI (New England Biolabs, Beverly, MA, USA), and inserted into pET28a (+) (Novagen, Madison, WI, USA) to add a His_6_-tag at the C-terminus of the recombinant protein. The expression plasmid was propagated in *Escherichia coli* DH5α (Toyobo), purified with the FastGene Plasmid Mini Kit (Nippon Genetics Co., Ltd.), and verified by sequencing (Operon Biotechnologies, Tokyo, Japan).

### Site-directed Mutagenesis

Site-directed mutagenesis was carried out by the method of Braman *et al.* [28] but using the KOD-plus DNA polymerase. The pET28a plasmid carrying the *bsel_2816* gene was used as template for PCR to obtain the DNA fragment containing the mutation. Pairs of the mutagenic primers (5′-cagggtcctgatgcataccatgagaacg-3′ for E475A, 5′-gttcagggtcctgatcaataccatgagaac-3′ for E475Q, and the complementary strands) were used. The parental strands in the PCR products were digested with DpnI, which specifically digests methylated and hemimethylated DNA, and the amplified DNA were retained. The nicked plasmid DNA incorporating the desired mutation was transformed into the *E. coli* DH5α. The expression plasmid for the mutants was purified with the FastGene Plasmid Mini Kit (Nippon Genetics Co., Ltd.), and verified by sequencing (Operon Biotechnologies).

### Expression and Purification of Recombinant Proteins

*E. coli* BL21 (DE3) (Novagen) transformants harboring the expression plasmid for Bsel_2816 or its mutants were grown at 37°C in 200 ml of Luria-Bertani medium (1% tryptone, 0.5% yeast extract, and 0.5% NaCl) containing 50 µg/ml of kanamycin up to an absorbance (Abs) of 0.6 at 600 nm. The expressions were induced by 0.1 mM isopropyl β-d-thiogalactopyranoside and continued at 18°C for 24 h. Wet cells, harvested by centrifugation at 20,000×*g* for 20 min, were suspended in 50 mM HEPES–NaOH buffer (pH 7.0) containing 500 mM NaCl (buffer A) and 10% glycerol. The suspended cells were disrupted by sonication (Branson sonifier 250A; Branson Ultrasonics, Emerson Japan, Ltd., Kanagawa, Japan) and the supernatant, collected by centrifugation at 20,000×*g* for 20 min, was applied to a HisTrap HP column (GE Healthcare, Buckinghamshire, UK) equilibrated with buffer A containing 10 mM imidazole using an ÄKTA prime system (GE Healthcare). After a wash with buffer A containing 22 mM imidazole and the following elution using a 22−400 mM imidazole linear gradient in buffer A, the fractions containing the recombinant protein (Bsel_2816) were pooled, dialyzed against 10 mM HEPES–NaOH buffer (pH 7.0), and concentrated (AMICON Ultra-15 filter; Millipore Co., Billerica, MA, USA). The concentration of the wild-type, E475Q mutant, and E475A mutant was determined spectrophotometrically at 280 nm using a theoretical extinction coefficient of ε = 124,110 M^−1^cm^−1^ on the basis of amino acid sequences [29]. The molecular masses of the purified proteins was estimated by SDS-PAGE (Mini-PROTEAN Tetra electrophoresis system; Bio-Rad Laboratories, Inc., Hercules, CA, USA) and by gel filtration (HiLoad 26/600 Superdex 200 pg; GE Healthcare) equilibrated with 10 mM HEPES–NaOH buffer (pH 7.0) containing 150 mM NaCl at the flow rate of 0.5 ml/min and using the marker proteins for molecular weight determination on High Pressure Liquid Chromatography (HPLC) (Oriental Yeast Co., Ltd., Tokyo, Japan) as standards.

### Thin Layer Chromatography (TLC)

TLC was performed on a TLC plate (Kieselgel 60 F254; Merck, Darmstadt, Germany). After spotting the samples, the plate was developed once with acetonitrile–water (4∶1 v/v) or twice with acetonitrile–water (9∶1 v/v). The TLC plates were then soaked in 5% sulfuric acid–methanol solution and heated in an oven until the spots of the carbohydrates became sufficiently visible.

### Enzymatic Quantification of Glc and βGlc1*P*

The concentration of Glc was determined by using a glucose oxidase/peroxidase kit, the Glucose C-II Test Wako. Two volumes of the coloring reagent of the kit were added to one volume of the sample. The increase in the Abs_505_ was measured after incubation for 60 min at 37°C. The concentration of βGlc1*P* was determined by using the βPGM/G6PDH method [30] with the working reagent containing 5 U/ml βPGM, 5 U/ml G6PDH, 4 mM thio-NAD^+^, 20 µg/ml Glc16b*P*, and 10 mM MgCl_2_ in 50 mM MOPS–NaOH buffer (pH 7.0). An aliquot of sample was mixed with the same volume of the working reagent and the increase in the Abs_400_ was measured after incubation for 60 min at 37°C.

### Cleavage of α-linked Glucobioses

To examine the specificity on α-linked glucobioses, each reaction mixture containing each disaccharide (10 mM; kojibiose, nigerose, maltose, isomaltose, or trehalose), 10 mM inorganic phosphate (Pi), and 29 µM Bsel_2816 in 50 mM MES–NaOH buffer (pH 6.0) was incubated at 30°C for 3 h. The products were analyzed by TLC. The reaction rate for the cleavage of kojibiose was determined by quantifying the Glc liberated in the reaction mixtures containing various concentrations of kojibiose (1–10 mM) and Pi (0–10 mM) in 40 mM sodium acetate buffer (pH 5.5) at 30°C. An aliquot of the reaction mixture was mixed with the same volume of dimethyl sulfoxide to stop the enzymatic reaction, followed by the enzymatic quantification of Glc. The kinetic parameters were calculated by curve-fitting the experimental data to the theoretical equation (i) for sequential Bi Bi mechanism (A = kojibiose, B = Pi) using GraFit version 7.0.2 (Erithacus Software Ltd., London, UK).
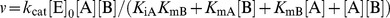
(i)


#### Acceptor specificity analysis

To determine the acceptor specificity of Bsel_2816 in the reverse phosphorolysis, each reaction mixture containing 10 mM each acceptor and 10 mM βGlc1*P* in 50 mM MES–NaOH buffer (pH 6.0) was reacted with Bsel_2816 at 30°C. After the reaction, the products were analyzed by TLC.

### Preparation and Structural Determination of the Product from Glycerol

A reaction mixture (5 ml) containing 500 mM βGlc1*P* (disodium salt buffered at pH 6.0 with HCl), 1 M glycerol, and 0.25 mg/ml Bsel_2816 was incubated at 30°C for 18 h. After terminating the reaction by boiling, an aliquot (3 ml) was deionized with Amberlite MB-3 (Organo, Tokyo, Japan), applied on an Amberlite IRA400 column (OH^−^ type, 40 ml), and eluted with H_2_O to remove the reducing sugars. The fractions containing the product were collected, concentrated to 9 ml, and loaded on a Toyopearl HW40F column (50 mm internal diameter×800 mm; Tosoh, Tokyo, Japan) equilibrated with distilled water. The product was eluted by distilled water at a flow rate of 5 ml/min. The fractions containing the product were collected, concentrated, and lyophilized to be syrup (298 mg). The one-dimensional (^1^H and ^13^C) and two-dimensional [double-quantum-filtered correlation spectroscopy (DQF-COSY), heteronuclear single-quantum coherence (HSQC), and heteronuclear multiple-bond correlation (HMBC)] NMR spectra of the product were recorded in D_2_O with 2-methyl-2-propanol as an internal standard at 298 K using the Bruker Avance 500 spectrometer (Bruker Biospin, Rheinstetten, Germany). The product was determined to be 2-*O*-α-d-glucosylglycerol (GG).

### Measurement of Enzyme Activity

The phosphorolytic activity of GG was determined by quantifying βGlc1*P* produced during the enzymatic reaction from 10 mM GG and 10 mM Pi in 40 mM HEPES–NaOH buffer (pH 8.0) at 30°C. An aliquot of the reaction mixture was mixed with the same volume of dimethyl sulfoxide to stop the enzymatic reaction, followed by the enzymatic quantification of βGlc1*P*. The hydrolytic activity on βGlc1*P* was determined by measuring the increase in Glc released during the enzymatic reaction from 10 mM βGlc1*P* in 40 mM MES–NaOH buffer (pH 6.0) or 40 mM HEPES–NaOH buffer (pH 7.5) at 30°C. An aliquot of the reaction mixture was mixed with the same volume of dimethyl sulfoxide to stop the enzymatic reaction, followed by the enzymatic quantification of Glc. The synthetic activity of GG was determined by measuring the increase in Pi in the reaction mixture containing 10 mM βGlc1*P* and 10 mM glycerol in 40 mM HEPES–NaOH buffer (pH 7.5) at 30°C by using the method of Lowry and Lopez [31].

### Temperature and pH Profile

The effects of pH on the phosphorolytic, hydrolytic, and synthetic activities of 212 nM Bsel_2816 were measured under the standard conditions by substituting each 40 mM reaction buffer into the following 40 mM buffers: sodium citrate (pH 3.0–5.5), Bis(2-hydroxyethyl)iminotris(hydroxymethyl)methane–HCl (pH 5.5–7.0), HEPES–NaOH (pH 7.0–8.5), and glycine-NaOH (pH 8.5–10.5). The thermal and pH stabilities were evaluated by measuring the residual synthetic activity under the standard conditions after the incubation of Bsel_2816 (7 µM and 11 µM, respectively) at temperatures 30–90°C for 15 min in 10 mM HEPES–NaOH buffer (pH 7.0) containing 0.1% bovine serum albumin and at the various pH values at 4°C for 24 h, respectively.

### The Kinetic Parameters of Bsel_2816

A kinetic analysis of the phosphorolytic reaction of GG was performed using the continuous βGlc1*P* assay. The reaction mixtures (150 µl) were prepared in the wells of a 96-well microtiter plate with various concentrations of GG (1.0–20 mM) and Pi (0.5–10 mM), and 5.2 nM Bsel_2816 in 40 mM HEPES–NaOH buffer (pH 8.0) containing 2.5 U/ml βPGM, 2.5 U/ml G6PDH, 2 mM thio-NAD^+^, 5 µg/ml Glc16b*P*, and 5 mM MgCl_2_. The reaction was carried out in a temperature-controlled microplate reader (Multiskan GO, ThermoFisher Scientific, Waltham, MA) at 30°C, and the Abs_400_ was continuously monitored at 30-s intervals without stopping the reaction. The kinetic parameters were calculated by curve-fitting the experimental data to the theoretical equation (i) for sequential Bi Bi mechanism (A = GG, B = Pi) using the GraFit version 7.0.2.

The initial velocities of the hydrolytic reaction on βGlc1*P* were determined under the standard conditions with 5.2 nM Bsel_2816 and various concentrations of βGlc1*P* (0.1–10 mM). The kinetic analysis of the synthetic reaction with glycerol as acceptor was carried out under the standard conditions (5.2 nM Bsel_2816) and various concentrations of glycerol (1.0–100 mM) or βGlc1*P* (ranging from 0.1 to 10 mM) with 10 mM or 100 mM of each opposite substrate, respectively. The kinetic parameters were calculated by curve-fitting the experimental data with the Michaelis–Menten equation (ii) using GraFit version 7.0.2.
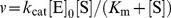
(ii)


The inhibition of the hydrolysis of βGlc1*P* by glycerol was examined in the reaction mixture containing 10 mM βGlc1*P* and various concentrations of glycerol (0–100 mM) in 40 mM HEPES–NaOH buffer (pH 7.5) and the increase in the concentration of Glc was quantified. The rate of the release of Glc was regressed with the concentration of glycerol to determine the dissociation constant of glycerol using the equation (iii) with GraFit version 7.0.2.
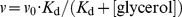
(iii)


### Position of Hydrolysis in βGlc1*P*

βGlc1*P* bis(cyclohexylammonium) salt (46 mg, 0.10 mmol) was dissolved in 1 ml of H_2_O and applied to a Dowex 50 column (H^+^ form, Wako Pure Chemicals) to remove the counter-ion (cyclohexylamine). An appropriate amount of 1 M NaOH was added to the decationized solution containing free βGlc1*P* (5 ml) adjusting pH to 6.0, followed by concentration to 1 ml by a centrifuge evaporator to obtain 100 mM βGlc1*P*–Na buffer (pH 6.0). An aliquot of the 100 mM βGlc1*P*–Na buffer (20 µl) was mixed with 70 µl of H_2_^18^O (97%, Sigma-Aldrich) or unlabeled H_2_O as a control. The hydrolysis of βGlc1*P* was started by adding 10 µl of 29 µM Bsel_2816 in 4 mM MES–NaOH buffer (pH 6.0) to the solution. After the incubation at 30°C for 4 h, βGlc1*P* was completely hydrolyzed into Glc and Pi. The solution was lyophilized, dissolved in 50% methanol, and subjected to negative and positive ESI-MS analysis by using a Bruker APEX II 70e Fourier transformed cyclotron resonance mass spectrometer (Bruker Daltonics, Billerica, MA, USA).

### Specificity on the Anomeric Configuration

The anomeric configuration of the glucose generated during the hydrolysis of βGlc1*P* was analyzed by HPLC [32], using an HPLC system (Prominence, Shimadzu, Kyoto, Japan) equipped with a refractive index detector, RID10A (Shimadzu). The hydrolysis of βGlc1*P* was performed in the reaction mixture containing 50 mM βGlc1*P* and 13.8 µM Bsel_2816 prepared in 20 mM MES–NaOH buffer (pH 6.0) at 30°C. After the reaction for 1.5 and 25 min, aliquots (10 µl each) of the reaction mixtures were immediately injected into the HPLC and separated with a TSK amide-80 column (4.6 mm internal diameter×250 mm; Tosoh, Tokyo, Japan) by isocratic mode with 80% acetonitrile at a flow rate of 1.5 ml/min.

## Results

### Gene Cloning, Expression, and Purification of Recombinant Bsel_2816

**Figure 1:**
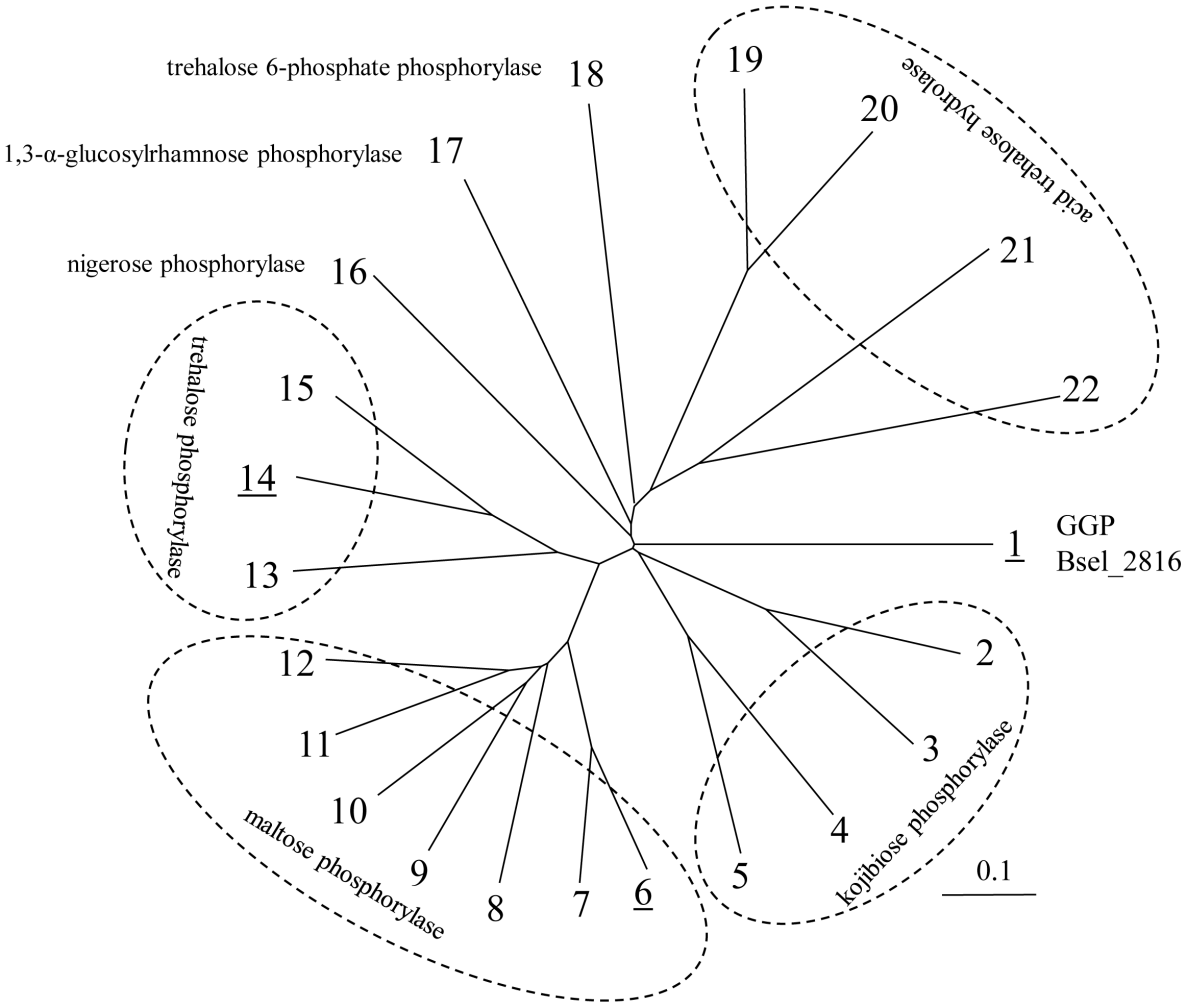
Phylogenetic tree of the GH65 enzymes characterized. Sequences used are: 1, Bsel_2816 of *B. selenitireducens* MLS10 (GenBank ID: ADI00307.1); 2, All1058 of *Nostoc* sp. PCC 7120; 3, All4989 of *Nostoc* sp. PCC 7120; 4, Csac_0439 of *Caldicellulosiruptor saccharolyticus* DSM 8903 (ABP66077.1); 5, KojP of *Thermoanaerobacter brockii* ATCC 35047 (AAE30762.1); 6, Bsel_2056 of *B. selenitireducens* MLS10 (ADH99560.1); 7, MapA of *Paenibacillus* sp. SH-55 (BAD97810.1); 8, maltose phosphorylase of *Lactobacillus sanfranciscensis* DSM 20451 (CAA11905.1); 9, MPase of *Bacillus* sp. RK-1 (BAC54904.1); 10, EF0957 of *Enterococcus faecalis* V583 (AAO80764.1); 11, LBA1870 of *Lactobacillus acidophilus* NCFM (AAV43670.1); 12, maltose phosphorylase from *Lactobacillus brevis* ATCC 8287 (Q7SIE1); 13, TreP of *T. brockii* ATCC 35047 (AAE18727.1); 14, Bsel_1207 of *B. selenitireducens* MLS10 (ADH98720.1); 15, TPase of *Geobacillus stearothermophilus* SK-1 (BAC20640.1); 16, Cphy_1874 of *Clostridium phytofermentans* ISDg (ABX42243.1); 17, Cphy_1019 of *C. phytofermentans* ISDg (ABX41399.1); 18, TrePP of *Lactococcus lactis* subsp. *lactis* Il1403 (AAK04526.1); 19, Atm1 of *Metarhizium acridum* CQMA102 (ABB51158.1); 20, TreA of *Aspergillus nidulans* FGSCA4 (EAA66407.1); 21, Ath1 of *Saccharomyces cerevisiae* S288c (CAA58961.1); 22, Atc1 of *Candida albicans* (AAV05390.1). Proteins from *B. selenitireducens* MLS10 are underlined.

The phylogenetic tree analysis showed that Bsel_2816 is distantly related with the kojibiose phosphorylase and nigerose phosphorylase (Fig. 1). In this study, the *bsel_2816* gene cloned from the genomic DNA of *B. selenitireducens* MLS10 was expressed as a His_6_-tag fusion protein in *E. coli* BL21 (DE3) to investigate its enzymatic properties. Purified protein (approximately 14 mg) was obtained from the cell lysate of 200 ml culture. The purified Bsel_2816 migrated in SDS-PAGE as a single protein band with an estimated molecular mass of approximately 87 kDa, in agreement with the theoretical molecular weight of 86,259. However, this native molecular mass was determined to be 174 kDa by gel filtration, suggesting that Bsel_2816 was a homodimeric enzyme.

### Examination of the Phosphorolytic Activity on the Substrates of the known GH65 Phosphorylases

**Figure 2:**
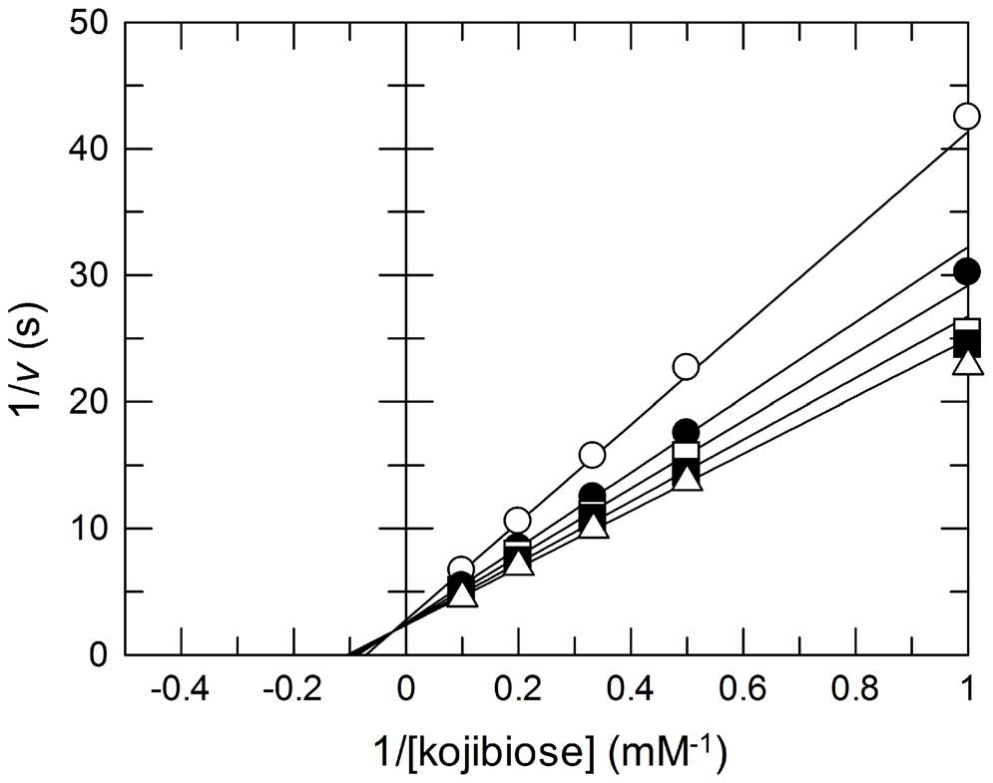
Double-reciprocal plot of the Pi-dependent hydrolysis of kojibiose by Bsel_2816 at various concentrations of Pi. Open circles, 1 mM Pi; closed circles, 2 mM Pi; open squares, 3 mM Pi; closed squares, 5 mM Pi; and open triangles, 10 mM Pi. The kinetic parameters were *k*_cat_ = 0.43 ± 0.02 (s^−1^), *K*_mA_ = 8.9 ± 0.6 (mM), *K*_mB_ = 0.19 ± 0.12 (mM), and *K*_iA_ = 41 ± 29 (mM). The values were determined by regressing the data with the following equation using the Grafit Version 7.02: *v* = *k*_cat_[E]_0_[A][B]/(*K*_iA_ *K*_mB_+*K*_mA_[B]+*K*_mB_[A]+[A][B]).

The phosphorolytic activity of Bsel_2816 on α-linked glucobioses (i.e., kojibiose, nigerose, maltose, isomaltose, and trehalose) including the known substrates of the GH65 enzymes was examined. Bsel_2816 cleaved kojibiose at a slow rate only in the presence of Pi. To our surprise, Glc was detected as the sole product and βGlc1*P* was not detected at all. The optimum pH for the cleavage of kojibiose was determined to be 5.5. The measurement of the reaction rate with various concentrations of kojibiose and Pi revealed that the reaction proceeded with a sequential Bi Bi mechanism, similarly to other inverting phosphorylases, with the following kinetic parameters: *K*_mA_, 8.9 mM; *K*_iA_, 41 mM; *K*_mB_, 0.19 mM; *k*_cat_ 0.43 s^−1^, where A and B represent kojibiose and Pi, respectively (Fig. 2). The reaction mechanism suggests that Pi was involved in the cleavage of kojibiose even though no βGlc1*P* was detected in the products.

### Hydrolytic Activity on βGlc1*P*

To determine the substrates of Bsel_2816 as a GH65 phosphorylase, the reverse phosphorolysis was examined with various sugars [hexoses (d-glucose, d-mannose, d-allose, d-galactose, d-altrose, d-talose, and d-fructose), pentoses (d-xylose, d-lyxose, d-ribose, d-arabinose, and l-arabinose), aminosugars (d-glucosamine, d-galactosamine, *N*-acetyl-d-glucosamine, *N*-acetyl-d-mannosamine, *N*-acetyl-d-galactosamine), uronic acids (d-glucuronic acid, and d-galacturonic acid), deoxyhexoses (1,5-anhydro-d-glucitol, 2-deoxy-d-glucose, and l-rhamnose), derivatives of d-glucose (α-d-glucose 1-phosphate, d-glucose 6-phosphate, 3-*O*-methyl-d-glucose, methyl-α-d-glucoside, and methyl-β-d-glucoside), and disaccharides (trehalose, kojibiose, nigerose, maltose, isomaltose, sucrose, sophorose, laminaribiose, cellobiose, gentiobiose, lactose, lactulose, melibiose, xylobiose, β-1,4-mannobiose, and *N*,*N'*-diacetylchitobiose)] as acceptors and βGlc1*P* as the donor. No transfer product was detected on TLC with any sugar acceptors that were examined. However, the consumption of βGlc1*P* and the concomitant generation of Glc were detected with all the acceptors. The concomitant generation of Glc and Pi was detected during the reaction in the absence of the acceptor molecule, clearly indicating that Bsel_2816 hydrolyzed βGlc1*P*. The rates of the hydrolysis of 10 mM βGlc1*P* in the presence and in the absence of 10 mM Glc were identical. The optimum pH for the hydrolysis was determined to be 6.0. The kinetic parameters of the hydrolysis of βGlc1*P* at pH 6.0 and pH 7.5 were determined to be as follows: *K*_m_, 0.041 and 0.16 mM; *k*_cat_, 2.8 and 1.7 s^−1^, respectively.

### Position of the Hydrolysis in βGlc1*P*

**Figure 3:**
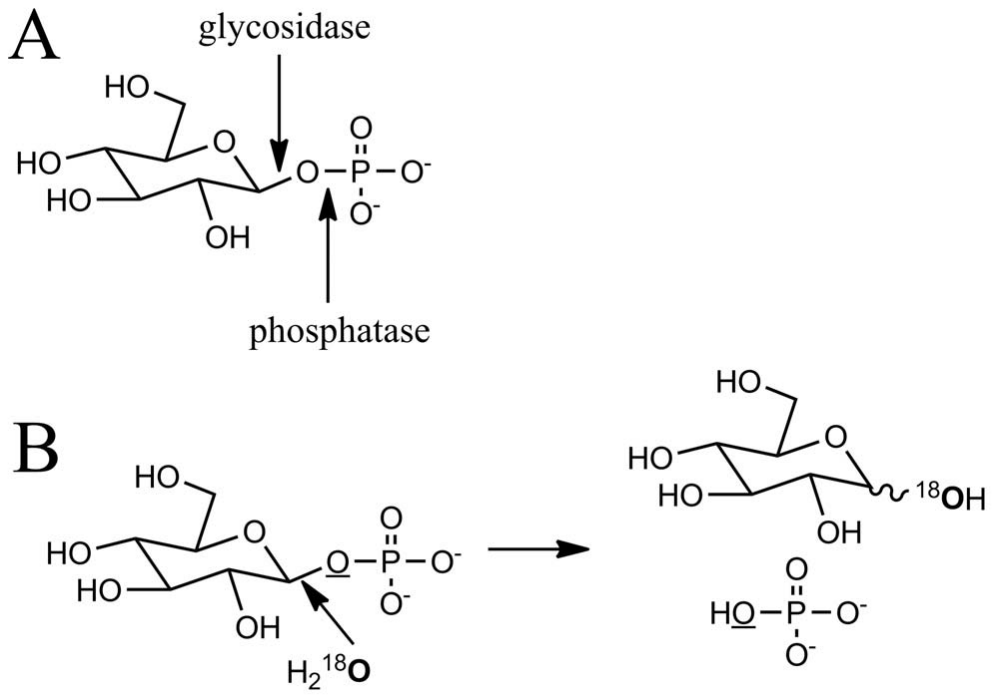
Linkages cleaved in the hydrolysis of βGlc1*P*. A: Cleavage positions by the action of phosphatases and glycosidases. B: Results of the enzymatic hydrolysis of βGlc1*P* by Bsel_2816 in H_2_^18^O.

The enzymatic hydrolysis of phosphate esters is generally catalyzed by phosphatases that cleave the PO bond of the phosphate ester [33]. To investigate the mechanism of the hydrolysis of βGlc1*P* by Bsel_2816, the incorporation of an oxygen atom was examined in ^18^O-labled water. In negative MS (Fig. S1A) of the control reaction, the ion signals at 194.95 *m/z* corresponding to [2H_3_PO_4_ − H]^−^ (calcd 194.947) and 277.03 *m/z* corresponding to [H_3_PO_4_ + Glc − H]^−^ (calcd 277.033) were detected. In the H_2_^18^O reaction, no signal of [2H_3_PO_4_ − H (^18^O)]^−^ (calcd 196.951) and [2H_3_PO_4_ − H (2^18^O)]^−^ (calcd 198.955) was detected, clearly indicating that ^18^O was not incorporated in the Pi during the hydrolysis. On the other hand, the signal at 277.03 *m/z* decreased and a new signal at 279.04 *m/z* corresponding to [H_3_PO_4_ + Glc − H (^18^O)]^−^ appeared, indicating that ^18^O was incorporated into either Pi or Glc. In positive MS (Fig. S1B), a signal at 203.05 *m/z* corresponding to [Glc+Na]^+^ (calcd 203.053) accompanied by a signal at 205.06 *m/z* corresponding to [Glc+Na(^18^O)]^+^ indicated that ^18^O was incorporated into Glc. It is concluded that the anomeric CO bond was cleaved during the hydrolysis of βGlc1*P*, indicating that the hydrolysis was a glycosidase-type reaction and not a phosphatase-type reaction (Fig. 3).

### The Anomeric Configuration of the Hydrolytic Product of βGlc1*P*

**Figure 4:**
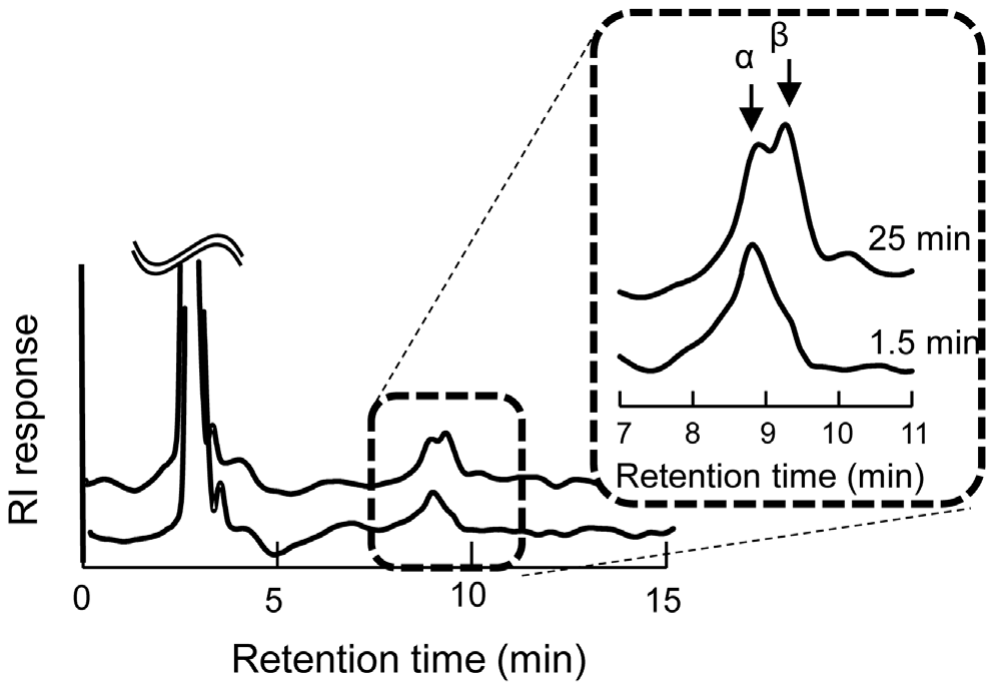
The anomeric specificity during the hydrolysis of βGlc1*P*.

The anomeric configuration of Glc generated through the hydrolysis of βGlc1*P* by Bsel_2816 was determined by HPLC (Fig. 4). The α-anomer of Glc was predominant at the initiation of βGlc1*P* hydrolysis, indicating that Bsel_2816 catalyzes the hydrolysis of βGlc1*P* with the inversion of the anomeric configuration. This result suggests that the hydrolysis was not due to a phosphatase-type reaction, in which the anomeric configuration of Glc should be retained during the hydrolysis. In addition, the anomeric inversion suggests that water molecules play a role as acceptors in the reverse phosphorolysis causing the hydrolysis.

### Determination of the Specificity of Bsel_2816

**Figure 5:**
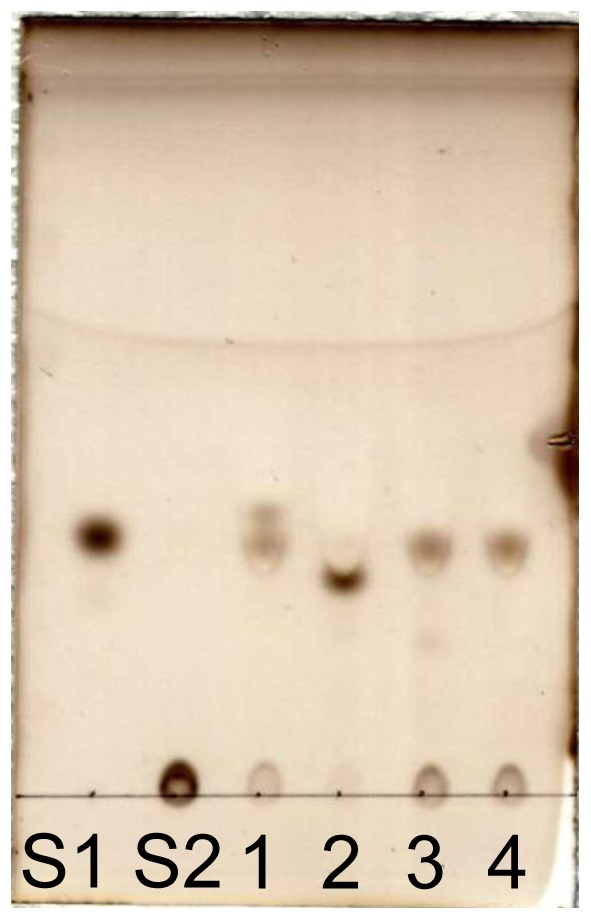
Screening for the suitable acceptor within polyols in the reverse phosphorolysis by Bsel_2816. The acceptors used are: 1, ethylene glycol; 2, glycerol; 3, erythritol; 4, no acceptor. S1 and S2, Glc and as βGlc1P as standards, respectively. The TLC plate was developed twice with acetonitrile-water (9∶1 v/v).

Because no acceptor was found within sugars, various alcohols (1-butanol, 2-butanol, cyclohexanol, benzylalcohol, hydroquinone, and phenol) and polyols (ethylene glycol, glycerol, erythritol, xylitol d-arabinitol, l-arabinitol, and d-glucitol) were used to identify the suitable acceptor. Only the hydrolysis of βGlc1*P* was detected with all the alcohols examined (data not shown). Among polyols, the consumption of βGlc1*P* was obviously faster with glycerol than with others (Fig. 5). A spot exhibiting slightly different *R_f_* value with Glc was detected on TLC. The synthetic reaction gave a single product from glycerol. The NMR spectra are shown in Fig. S2 and the assignment of the signals are summarized in Table 2. The *J*_1,2_ value of the Glc residue (3.9 Hz) indicates an α-d-glucoside. The HMBC cross signals indicate the glycosyl linkage with the position 2 of glycerol. Thus, the product was identified to be GG.

**Table 2:** Assignments of ^1^H and ^13^C NMR signals of 2-*O*-α-d-glucosylglycerol.

Residue	Position	^13^C-NMR	^1^H-NMR		
		δ (ppm)	δ (ppm)	pattern	*J* (Hz)
**glucose**	1	99.55	5.11	d	3.9
	2	73.21	3.54	dd	3.9, 9.9
	3	74.60	3.73	m	
	4	71.26	3.40	t	9.5
	5	73.67	3.83	m	
	6	62.18	3.83	m	
	6′		3.75	m	
**glycerol**	1	63.10	3.74	m	
	1′		3.74	m	
	2	**80.49**	**3.80**	m	
	3	62.04	3.76	dd	4.3, 12.1
	3′		3.70	dd	5.2, 12.1

Bold: HMBC cross signal is observed with the anomeric position of glucose.

**Figure 6:**
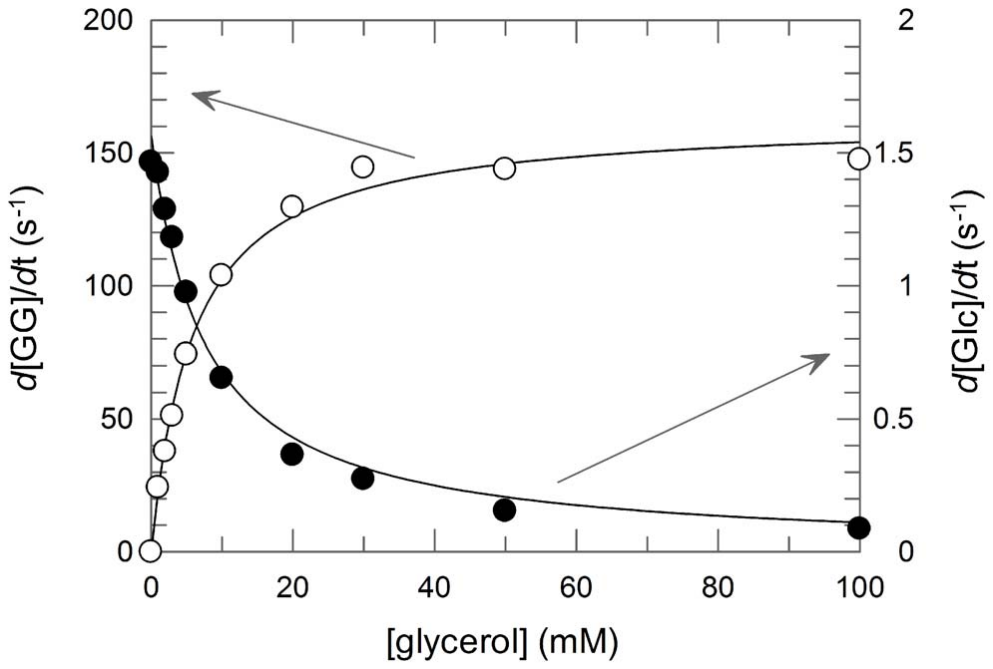
The reaction rates of the generations of GG and Glc from 10βGlc1*P* and various concentrations of glycerol. Open circles, *d*[GG]/*d*t; closed circles, *d*[Glc]/*d*t.

**Figure 7:**
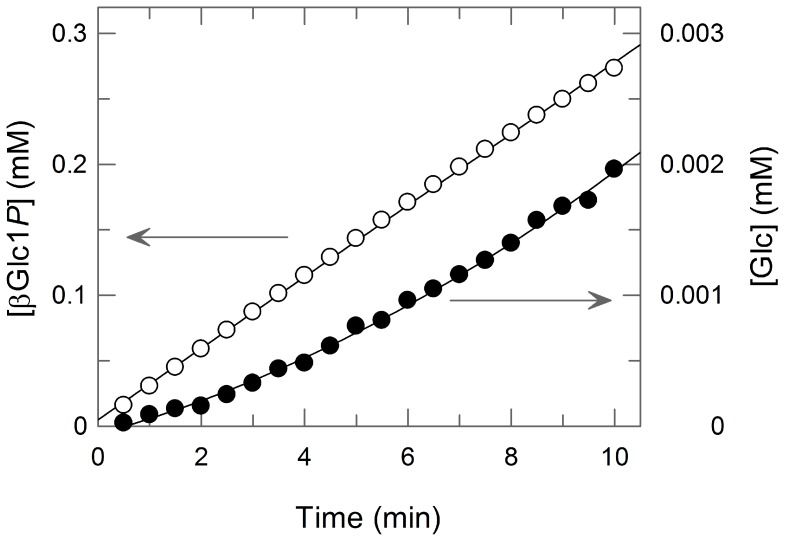
Time course of the increase of βGlc1*P* and Glc during the phosphorolysis of GG. Open circles, [βGlc1*P*]; closed circles, [Glc].

In the presence of glycerol, the synthesis of GG was much faster than the hydrolysis of βGlc1*P* (Fig. 6). In the presence of 10 mM glycerol, the hydrolysis rate was only 0.63% of that of the synthesis of GG. The hydrolysis was inhibited by glycerol (Fig. 6) with the *K*_d_ value of 7.6 mM. In addition, Bsel_2816 exhibited a weak reverse phosphorolysis activity on ethylene glycol, yielding two products (Glc and unknown) on TLC (Fig. 5). We confirmed the phosphorolysis of GG by the enzyme. During the phosphorolysis, the major product was βGlc1*P*, but a trace amount of Glc was detectable (Fig. 7). Herein, we propose 2-*O*-α-d-glucopyronosylglycerol: phosphate β-d-glucosyltransferase as the systematic name and 2-*O*-α-d-glucopyronosylglycerol phosphorylase (GGP) as the short name for Bsel_2816.

### Basic Properties of Bsel_2816

Bsel_2816 was stable up to 35°C during 15-min incubation, and it was stable at pH between 5.5−10.5 at 4°C for 24 h (data not shown). The optimum pHs for the phosphorolytic, synthetic, and hydrolytic reactions were pH 8.0, pH 7.5, and pH 6.0, respectively (data not shown).

### Kinetic Analyses of Bsel_2816

**Figure 8:**
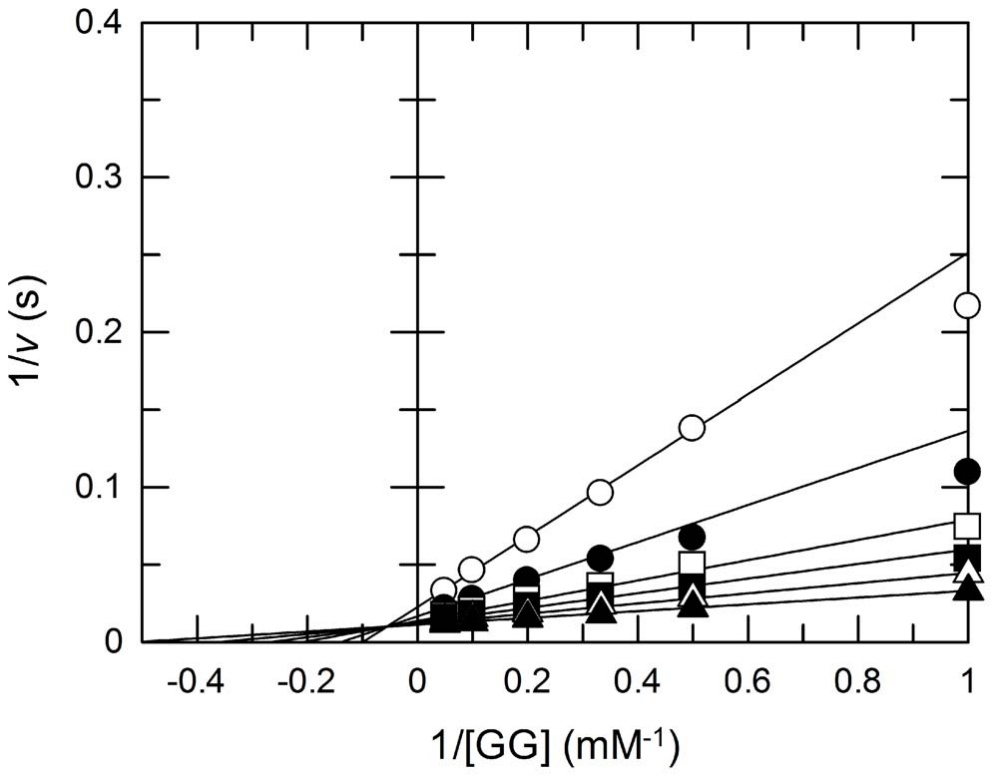
Double-reciprocal plot of the phosphorolysis of GG by Bsel_2816 at various concentrations of Pi. Open circles, 0.5; closed circles, 1 mM Pi; open squares, 2 mM Pi; closed squares, 3 mM Pi; open triangles, 5 mM Pi; and closed triangles, 10 mM Pi. The kinetic parameters were *k*_cat_ = 95±2 (s^−1^), *K*_mA_ = 1.1±0.1 (mM), *K*_mB_ = 0.57±0.07 (mM), and *K*_iA_ = 18±3 (mM). The values were determined by regressing the data with the following equation using Grafit Version 7.02: *v* = *k*_cat_[E]_0_[A][B]/(*K*_iA_ *K*_mB_+*K*_mA_[B]+*K*_mB_[A]+[A][B]).

The phosphorolytic reaction of GG was investigated at pH 8.0 at 30°C. Double reciprocal plots of the initial velocities against various initial concentrations of GG and P_i_ presented a series of lines intersecting at a point (Fig. 8), indicating that the phosphorolysis follows a sequential Bi Bi mechanism, as reported with other inverting phosphorylases [1], [17], [19], [20], [26], [34]–[39]. The kinetic parameters were calculated to be *K*_mA_, 1.1 mM; *K*_iA_, 18 mM; *K*_mB_, 0.57 mM; and *k*_cat_ 95 s^−1^, where A and B are GG and Pi, respectively. The parameters for GG are in the same ranges of those of the other inverting phosphorylases [1], [17], [19], [20], [26], [34]–[39], indicating that GG is the true substrate of Bsel_2816.

The kinetic parameters of the substrates in the synthetic reaction (glycerol and βGlc1*P*) were determined at pH 7.5. The *K*_m_ and *k*_cat_ values with glycerol were 5.7 mM and 163 s^−1^, respectively. The *K*_m_ value of glycerol agreed with the *K*_d_ value determined by the inhibition of hydrolysis of βGlc1*P* (7.6 mM). The *K*_m_ and *k*_cat_ values of βGlc1*P* were 0.45 mM and 180 s^−1^, respectively.

### Phosphorolytic and Hydrolytic Activities of Mutant Bsel_2816

Alignment of the amino acid sequences of GH65 phosphorylases suggests that E475 of Bsel_2816 is the general acid (for phosphorolysis)/base (for reverse phosphorolysis) catalyst [40], [41]. The phosphorolytic and hydrolytic activities of two mutants of Bsel_2816 at E475, E475A and E475Q, were less than 0.01% compared with those of the wild-type Bsel_2816, suggesting that both reactions were catalyzed by the same catalytic residue E475.

## Discussion

**Figure 9:**
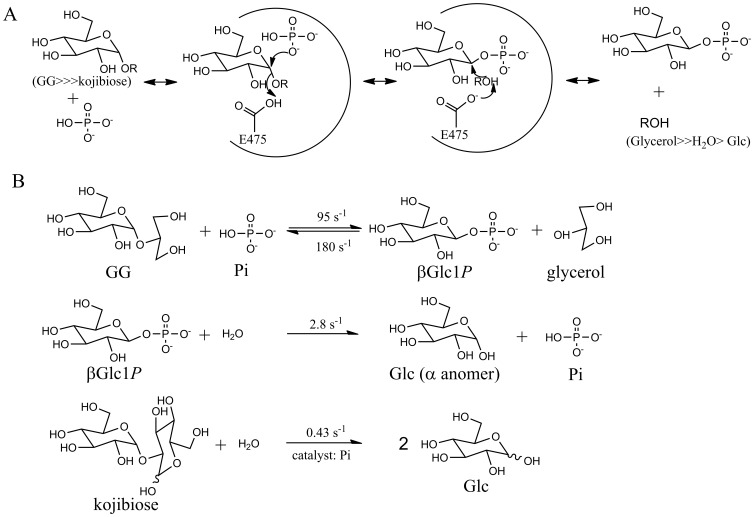
The reactions catalyzed by GGP. A, The reaction mechanism of GGP; B, Detectable reactions catalyzed by GGP.

We have determined that Bsel_2816 is a GGP. The reactions catalyzed by GG are summarized in Fig. 9. GGP is a unique phosphorylase in the following two characteristics.

First, any other phosphorylase reported exhibits its highest phosphorolytic activity on the glycosyl linkages bound with sugar residues (Table 1). Contrarily, GGP phosphorolyzes the glucosyl linkage bound with glycerol. Even though GG can be produced by other phosphorylases such as sucrose phosphorylase [42], [43], it is one of their minor substrates. The phosphorylases generally exhibit *K*_m_ values 1–10 mM with their main acceptors. Structural analyses of various phosphorylases have revealed that these enzymes recognize their acceptor molecules through their pyranose rings (or furanose ring in the case of sucrose phosphorylase) by a number of hydrogen bond interactions to obtain their strict acceptor specificities with the *K*_m_ values. The *K*_m_ value of GGP with glycerol was 5.7 mM, similarly to that of other phosphorylases, even though glycerol is a rather small molecule and does not adopt any rigid conformation.

Second, the other feature of GGP is the hydrolysis of βGlc1*P*. Other inverting phosphorylases do not hydrolyze sugar 1-phosphates [2] and this is the first report describing the significant hydrolysis by an inverting phosphorylase.

To investigate the mechanism of the hydrolysis, we determined the linkage cleaved in the hydrolysis, the anomeric configuration of the hydrolytic product, and the catalytic residue of the enzyme. Our results prove that the hydrolysis of βGlc1*P* occurs through the same catalytic mechanism as the reverse phosphorolysis, not through a phosphatase-type reaction. The hydrolysis is explainable as a glucosyl transfer reaction from βGlc1*P* to a water molecule with the anomeric inversion. The phosphate-dependent hydrolysis of kojibiose is not considered to be a direct hydrolysis. It is explained as follows: GGP phosphorolyzes kojibiose at a very slow rate to form βGlc1*P* that is immediately hydrolyzed to generate Glc and Pi by the same enzyme. In this mechanism, the α-anomer of Glc is considered to be generated during the hydrolysis, indicating a retaining hydrolysis. Unfortunately, the anomeric analysis during the hydrolysis of kojibiose was unsuccessful because of its very slow reaction. On the other hand, the hydrolytic reaction was much slower than the phosphorolysis and the synthesis of GG, suggesting that the hydrolytic activity may be negligible in its *in vivo* role.

Recently, indication of faint hydrolytic activity on α-mannnose 1-phosphate (αMan1*P*) by UhgbMP belonging to GH130, a β-1,4-mannooligosaccharide phosphorylase from unknown human gut bacterium, has been reported [44]. In the case, the enzyme at high concentration produced β-1,4-mannooligosaccharides with high degree of polymerization only from αMan1*P*, suggesting that the enzyme initially hydrolyzed αMan1*P* to generate mannose. The resultant mannose was not detected because it readily acted as the acceptor molecule to form β-1,4-mannobiose, which was a better acceptor than mannose to generate the polymer. This enzyme may share the same hydrolytic mechanism with GGP.

GG has been reported as a compatible solute for the control of the cytoplasmic osmotic pressure of cyanobacteria [45], [46]. Because *B. selenitireducens* is a halophilic bacterium, which is often isolated from soil with high concentration of salts [24], GG may possess a similar role in this bacterium. The biosynthetic pathway of the GG of cyanobacteria is explained with the concomitant reactions of glucosylglycerol-phosphate synthase (EC 2.4.1.213) [47] and glucosylglycerol phosphatase (EC 3.1.3.69) [48]. Other possible enzymes that form α-glucosyl linkage at the position 2 of the derivatives of glycerol are glucosyl-3-phosphoglycerate synthase (EC 2.4.1.266) [49] and glucosylglycerate synthase (EC 2.4.1.268) [50]. However, no homologous genes encoding these enzymes were found in the genomic sequence of *B. selenitireducens* MLS10 (GenBank ID: CP000896). The role of GGP is unclear but it may be involved in the biosynthesis of GG as well as in its cleavage. *B. selenitireducens* possesses potassium ion-dependent trehalose phosphorylase, even though the strain does not have either intracellular or extracellular biosynthetic pathways of trehalose [27]. *B. selenitireducens* has one more GH65 enzyme, maltose phosphorylase [26], which is a possible candidate to produce βGlc1*P*. These three enzymes may relate with the production of GG and trehalose in the cytoplasm of *B. selenitireducens* as solutes for the control of the cytoplasmic osmotic pressure.

GG is often isolated from the traditional Japanese food, fermented with “koji” (*Aspergillus oryzae*) [51]. It is valued for its application as a moisturizing agent and as external reagent protecting proteins against denaturation [43], [52]. The compound is commercially produced from maltose and glycerol using the transglycosylation activity of α-glucosidase as a mixture of α-glucosylglycerols [53], or from sucrose and glycerol by the action of sucrose phosphorylase [43], [54]. GGP is expected to be an alternative catalyst for the production of GG.

## Supporting Information

Figure S1 **ESI-MS spectra of the hydrolytic products from βGlc1** ***P*** **.** A, negative mode; B, positive mode.
(PDF)

Figure S2 **NMR spectra of the product from glycerol.** The product was identified to be 2-*O*-α-d-glucopyranosylglycerol. The spectra were taken in D_2_O at 298 K with 2-methyl-2-propanol as an internal standard using Bruker Avance 500 spectrometer. (A) ^1^H-NMR spectrum; (B) ^13^C-NMR (proton decupling and DEPT135); (C) DQF-COSY; (D) HSQC; (E) HMBC.
(PDF)
