# Supplementary material for: 2-O-α-D-Glucosylglycerol Phosphorylase from Bacillus selenitireducens MLS10 Possessing Hydrolytic Activity on β-D-Glucose 1-Phosphate
Source: PLoS One. 2014 Jan 22;9(1):e86548. doi: 10.1371/journal.pone.0086548 (PMC3899277; doi:10.1371/journal.pone.0086548)
Supplement: Figure S1 — ESI-MS spectra of the hydrolytic products from βGlc1 P . A, negative mode; B, positive mode. (PDF) [file pone.0086548.s001.pdf]

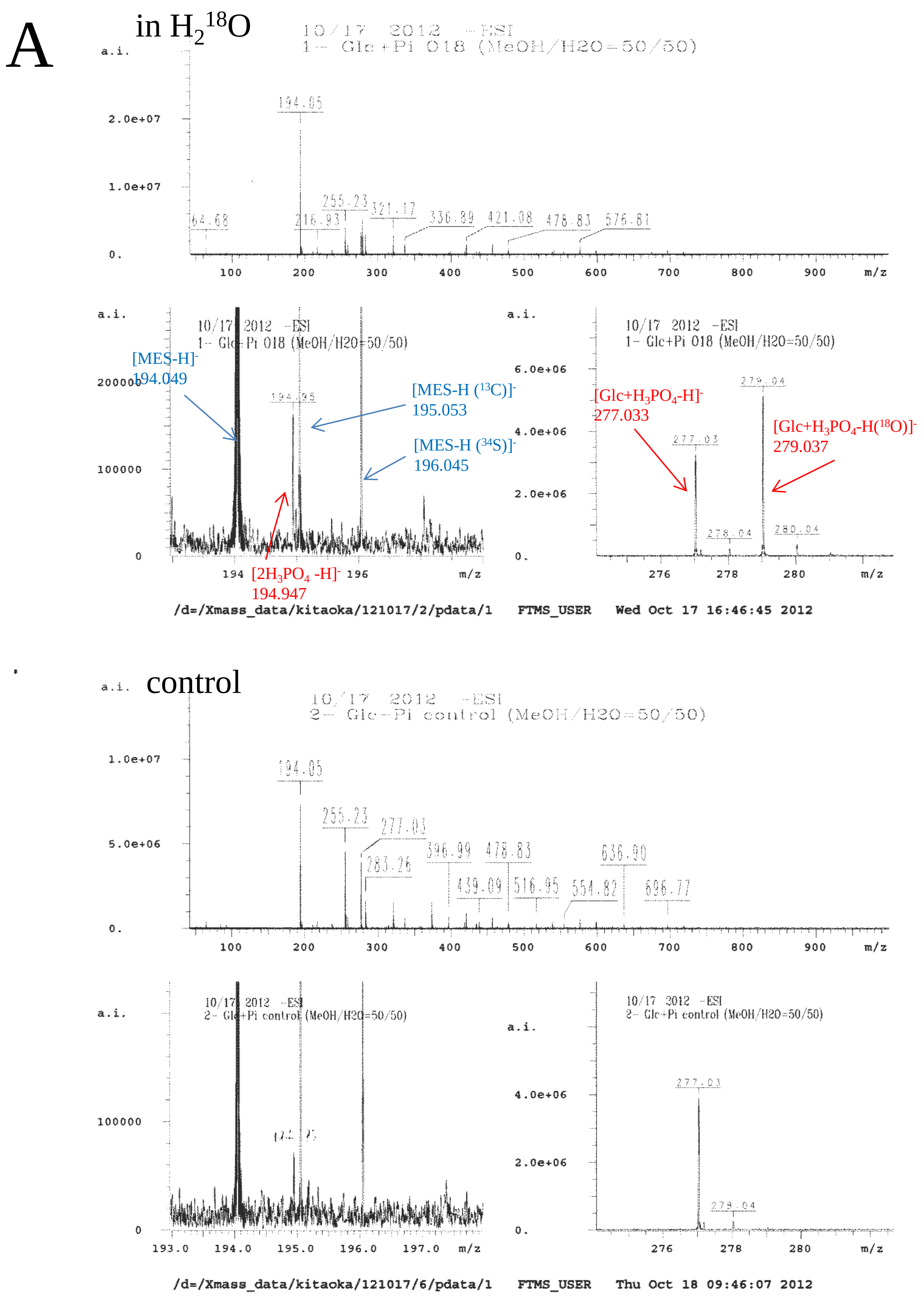

A
in $H_2^{18}O$
10/17 2012 -ESI
1- Glc+Pi O18 (MeOH/H2O=50/50)
a.i.
2.0e+07
1.0e+07
0.
194.05
255.23
321.17
164.68
216.93
336.89
421.08
478.83
576.81
100 200 300 400 500 600 700 800 900 m/z
[MES-H]-
194.049
[MES-H (13C)]-
195.053
[MES-H (34S)]-
196.045
[2H3PO4 -H]-
194.947
194.95
200000
100000
0
194 196 m/z
6.0e+06
4.0e+06
2.0e+06
[Glc+H3PO4-H]-
277.033
[Glc+H3PO4-H(18O)]-
279.037
279.04
277.03
278.04
280.04
276 278 280 m/z
/d=/Xmass_data/kitaoka/121017/2/pdata/1 FTMS_USER Wed Oct 17 16:46:45 2012
control
10/17 2012 -ESI
2- Glc+Pi control (MeOH/H2O=50/50)
1.0e+07
5.0e+06
194.05
255.23
277.03
283.26
396.99
478.83
439.09
516.95
554.82
636.90
696.77
100 200 300 400 500 600 700 800 900 m/z
100000
193.0 194.0 195.0 196.0 197.0 m/z
4.0e+06
2.0e+06
277.03
278.04
276 278 280 m/z
/d=/Xmass_data/kitaoka/121017/6/pdata/1 FTMS_USER Thu Oct 18 09:46:07 2012

B

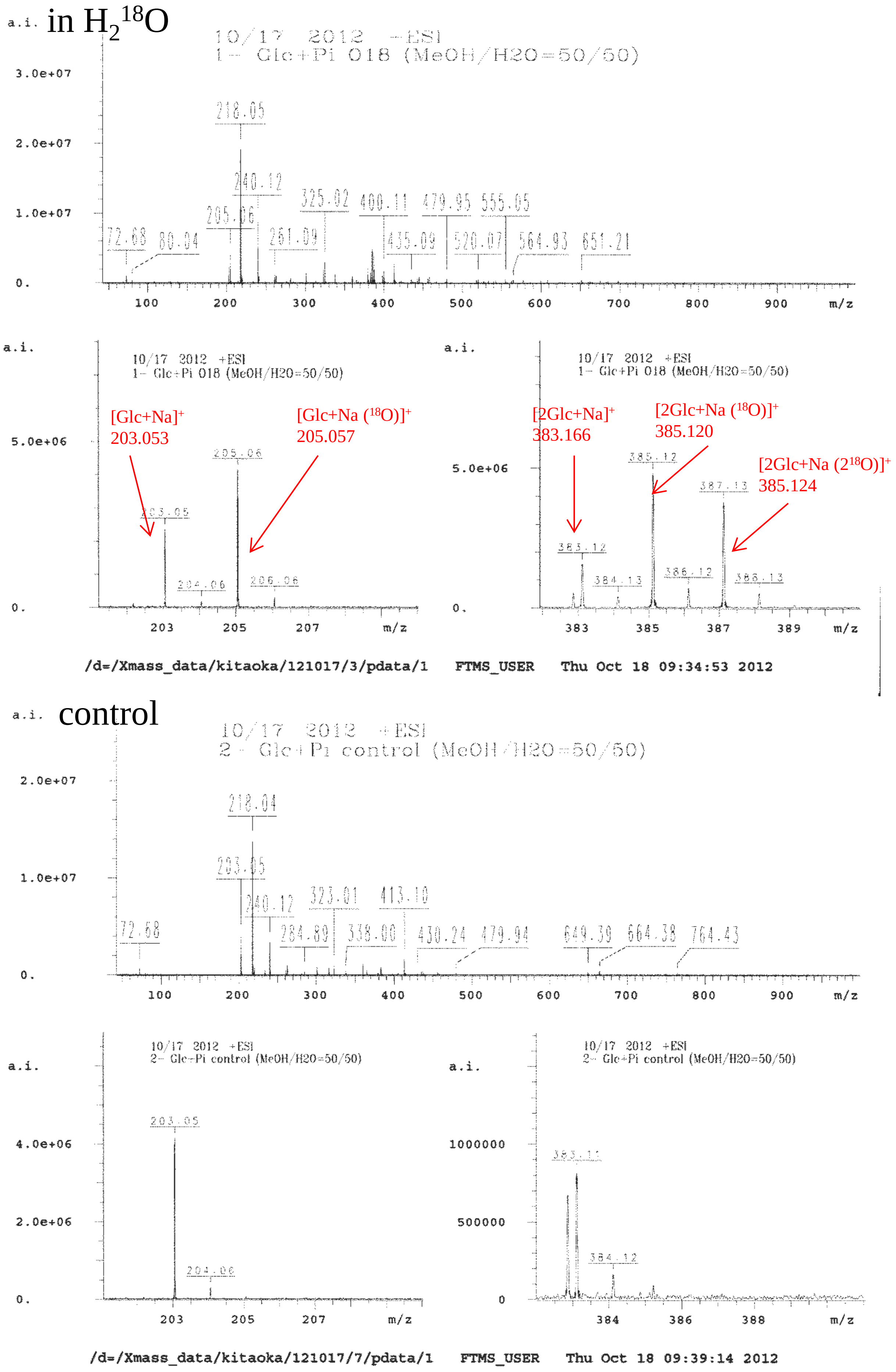

in $H_2{}^{18}O$
a.i.
10/17 2012 +ESI
1- Glc+Pi O18 (MeOH/H2O=50/50)
3.0e+07
2.0e+07
1.0e+07
0.
218.05
240.12
205.06
325.02
400.11
479.95
555.05
72.68
80.04
261.09
435.09
520.07
564.93
651.21
100
200
300
400
500
600
700
800
900
m/z
5.0e+06
[Glc+Na]+
203.053
[Glc+Na (18O)]+
205.057
205.06
203.05
204.06
206.06
203
205
207
[2Glc+Na]+
383.166
[2Glc+Na (18O)]+
385.120
[2Glc+Na (218O)]+
385.124
385.12
387.13
383.12
384.13
386.12
388.13
383
385
387
389
/d=/Xmass_data/kitaoka/121017/3/pdata/1 FTMS_USER Thu Oct 18 09:34:53 2012
control
10/17 2012 +ESI
2- Glc+Pi control (MeOH/H2O=50/50)
218.04
203.05
240.12
323.01
413.10
72.68
284.89
338.00
430.24
479.94
649.39
664.38
764.43
4.0e+06
2.0e+06
203.05
204.06
1000000
500000
0
383.11
384.12
384
386
388
/d=/Xmass_data/kitaoka/121017/7/pdata/1 FTMS_USER Thu Oct 18 09:39:14 2012
