# Supplementary material for: 2-O-α-D-Glucosylglycerol Phosphorylase from Bacillus selenitireducens MLS10 Possessing Hydrolytic Activity on β-D-Glucose 1-Phosphate
Source: PLoS One. 2014 Jan 22;9(1):e86548. doi: 10.1371/journal.pone.0086548 (PMC3899277; doi:10.1371/journal.pone.0086548)

Glcα1,2Glycerol

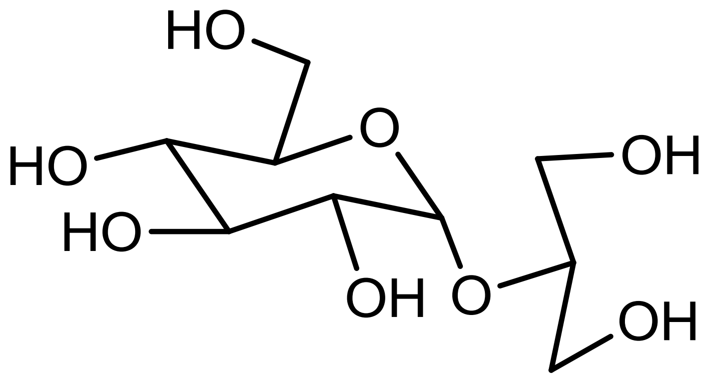

(A) ^{1}H-NMR

Glcα1,2Glycerol

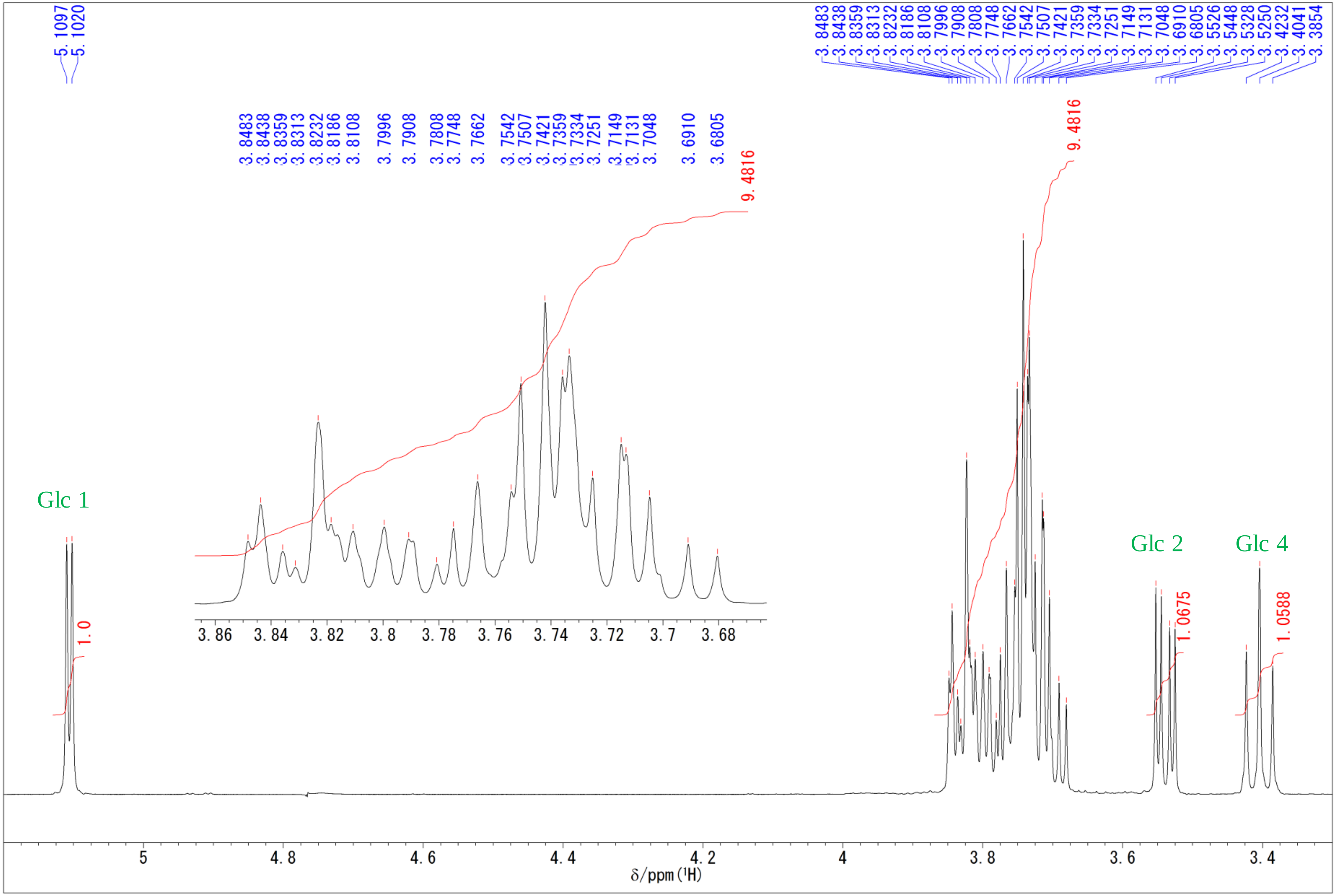

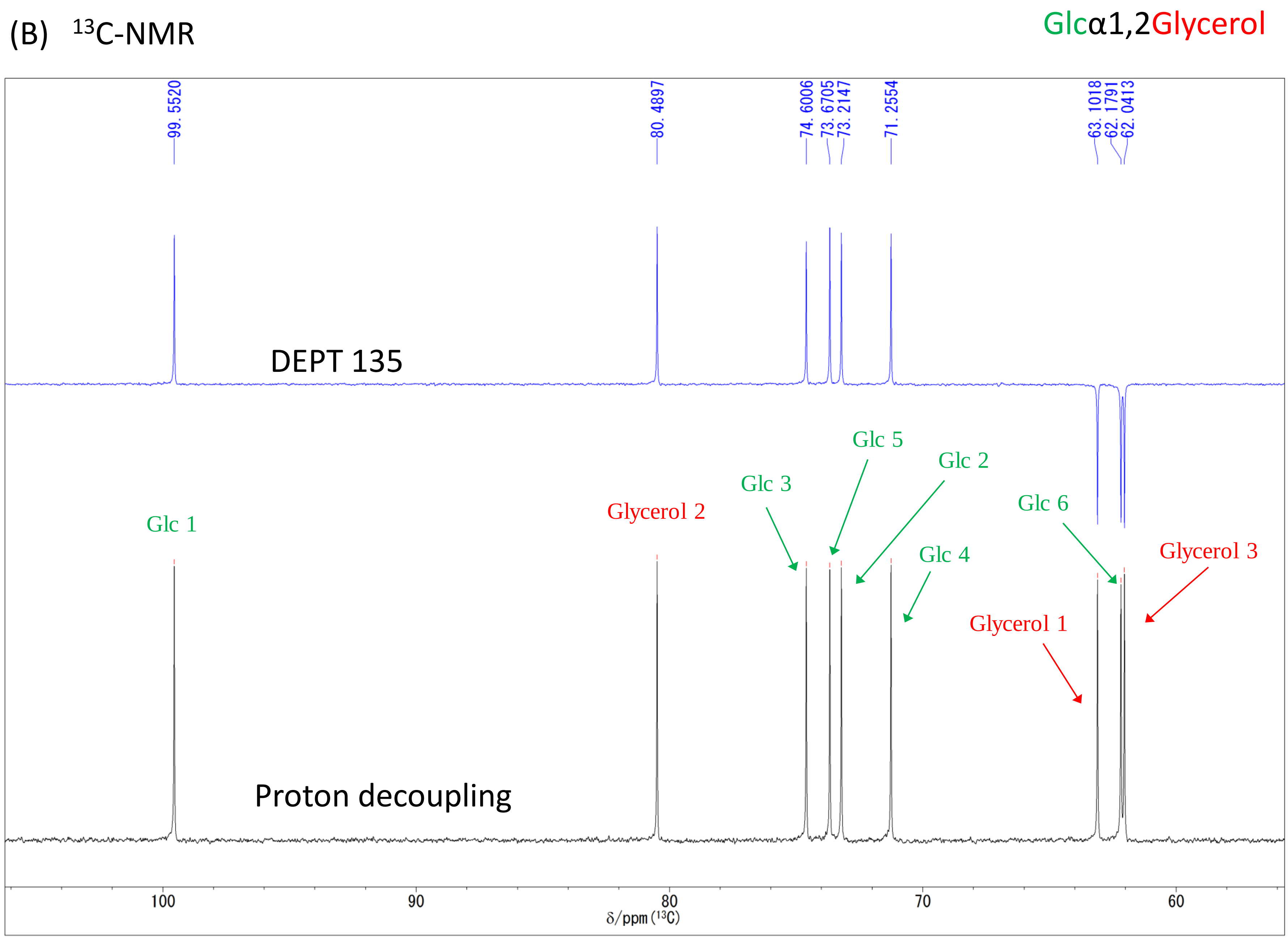

(B) 13C-NMR
Glcα1,2Glycerol
99. 5520
80. 4897
74. 6006
73. 6705
73. 2147
71. 2554
63. 1018
62. 1791
62. 0413
DEPT 135
Glc 1
Glycerol 2
Glc 3
Glc 5
Glc 2
Glc 4
Glc 6
Glycerol 3
Glycerol 1
Proton decoupling
100
90
80
70
60
δ/ppm (13C)

(C) DQF-COSY

Glcα1,2Glycerol

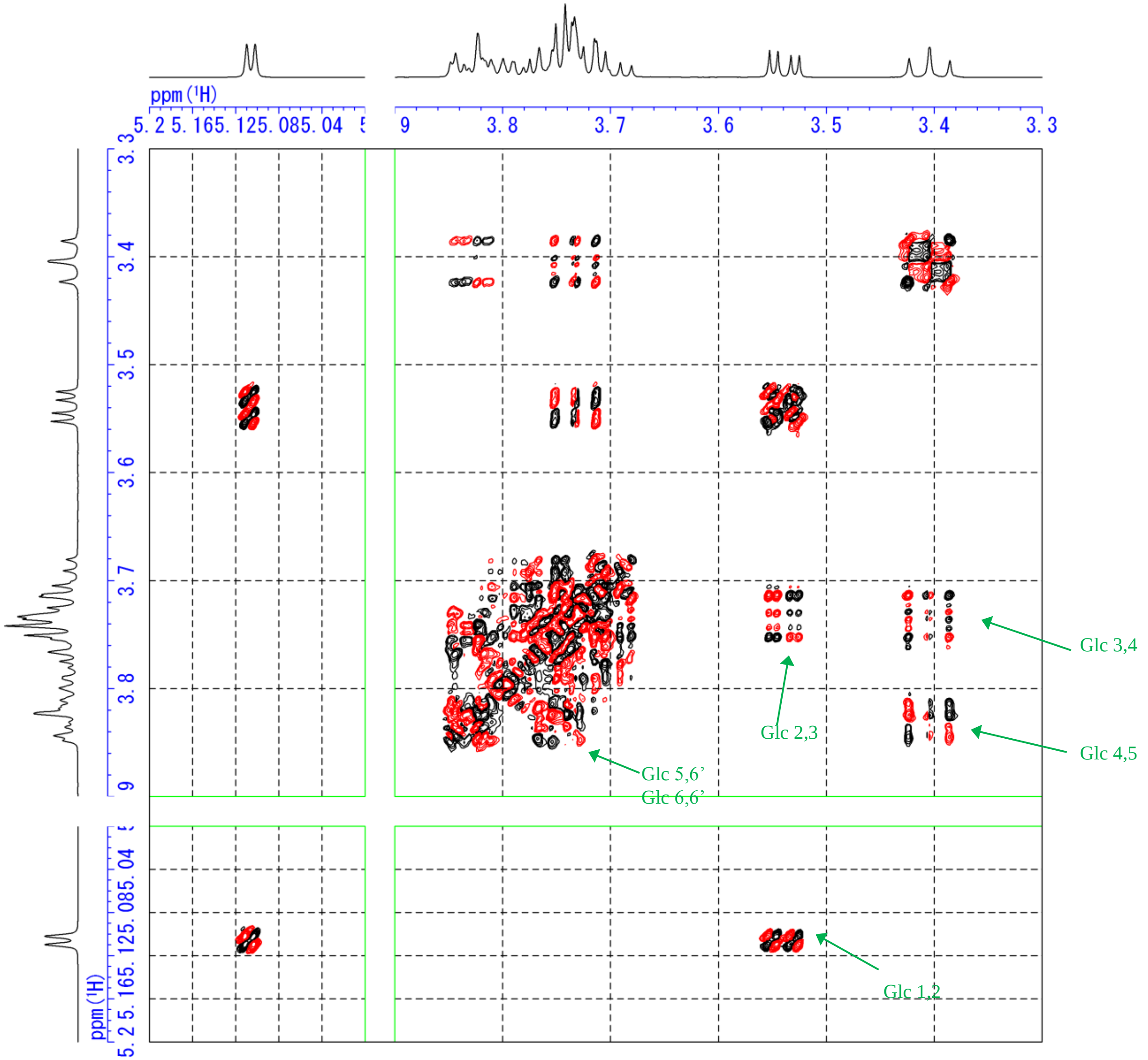

(D) HSQC

Glcα1,2Glycerol

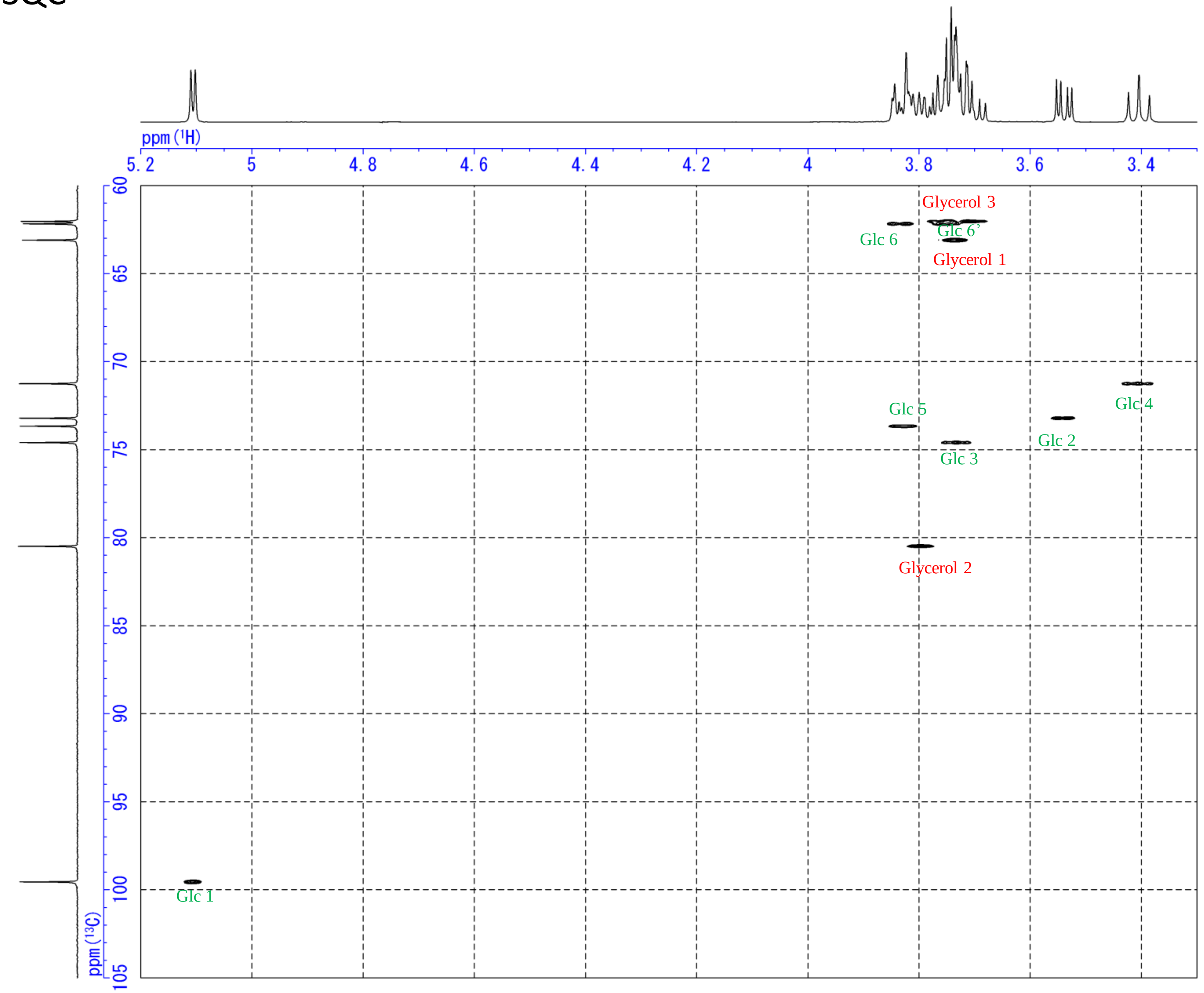

(E) HMBC

Glcα1,2Glycerol

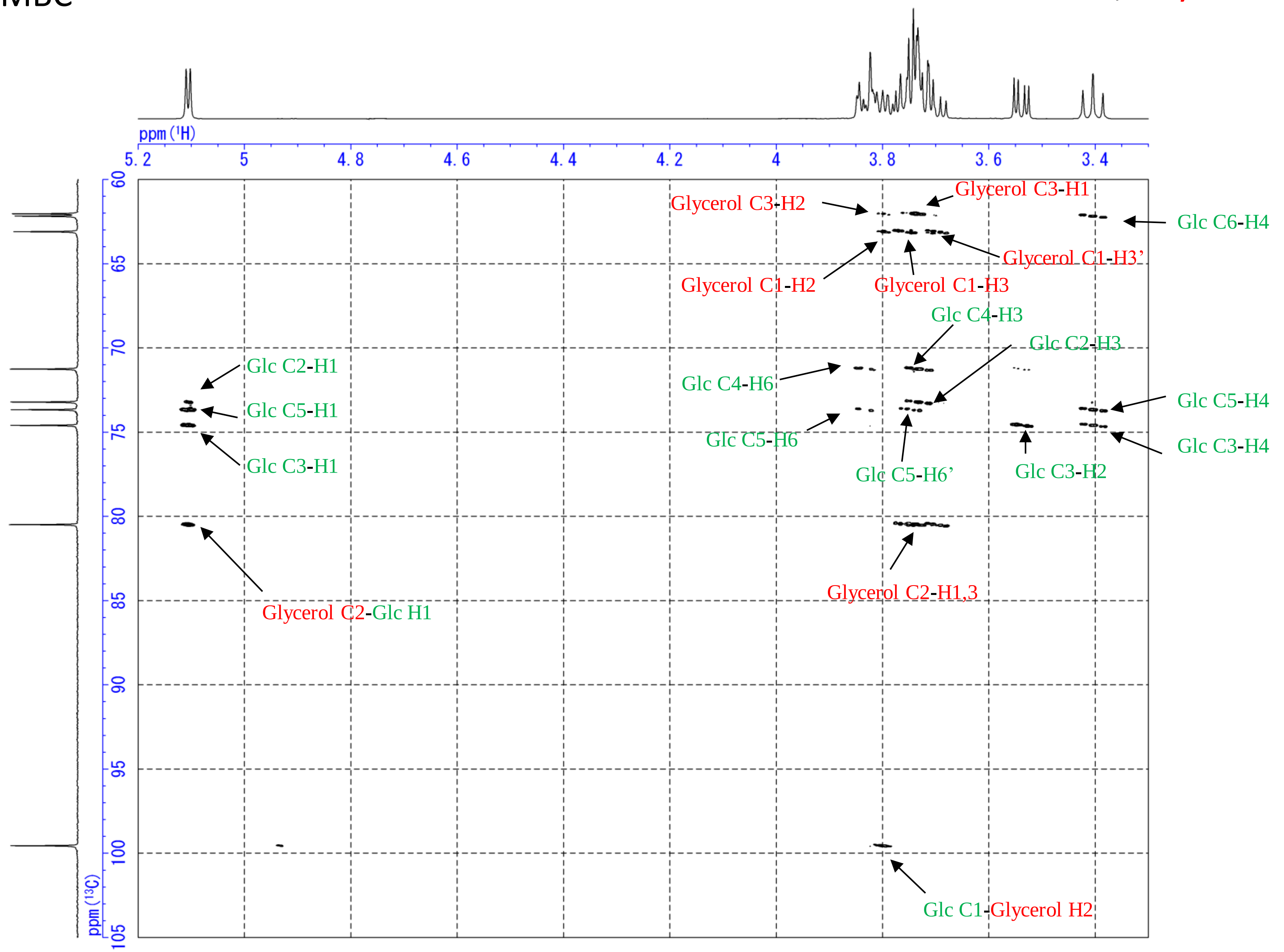

Supplement: Figure S2 — NMR spectra of the product from glycerol. The product was identified to be 2-O-α-d-glucopyranosylglycerol. The spectra were taken in D2O at 298 K with 2-methyl-2-propanol as an internal standard using Bruker Avance 500 spectrometer. (A) 1H-NMR spectrum; (B) 13C-NMR (proton decupling and DEPT135); (C) DQF-COSY; (D) HSQC; (E) HMBC. (PDF) [file pone.0086548.s002.pdf]
